# Prediction of 30-Day Readmission for COPD Patients Using Accelerometer-Based Activity Monitoring

**DOI:** 10.3390/s20010217

**Published:** 2019-12-30

**Authors:** Wen-Yen Lin, Vijay Kumar Verma, Ming-Yih Lee, Horng-Chyuan Lin, Chao-Sung Lai

**Affiliations:** 1 Department of Electrical Engineering, Center for Biomedical Engineering, Chang Gung University, Tao-Yuan 33302, Taiwan; d0421006@stmail.cgu.edu.tw; 2 Division of Cardiology, Department of Internal Medicine, Chang Gung Memorial Hospital, Tao-Yuan 33305, Taiwan; leemiy@mail.cgu.edu.tw; 3 Graduate Institute of Biomedical Engineering, Center for Biomedical Engineering, Chang Gung University, Tao-Yuan 33302, Taiwan; 4 Department of Thoracic Medicine, Chang Gung Memorial Hospital, Tao-Yuan 33305, Taiwan; lin53424@gmail.com; 5 Department of Electronic Engineering, Center for Biomedical Engineering, Chang Gung University, Tao-Yuan 33302, Taiwan; cslai@mail.cgu.edu.tw; 6 Department of Nephrology, Chang Gung Memorial Hospital, Linkou, Tao-Yuan 33305, Taiwan; 7 Department of Materials Engineering, Ming Chi University of Technology, New Taipei 24301, Taiwan

**Keywords:** accelerometers, actigraphy, activity monitoring, COPD, prediction, readmission risk, wearable devices

## Abstract

Chronic obstructive pulmonary disease (COPD) claimed 3.0 million lives in 2016 and ranked 3rd among the top 10 global causes of death. Moreover, once diagnosed and discharged from the hospital, the 30-day readmission risk in COPD patients is found to be the highest among all chronic diseases. The existing diagnosis methods, such as Global Initiative for Chronic Obstructive Lung Disease (GOLD) 2019, Body-mass index, airflow Obstruction, Dyspnea, and Exercise (BODE) index, modified Medical Research Council (mMRC), COPD assessment test (CAT), 6-minute walking distance, which are adopted currently by physicians cannot predict the potential readmission of COPD patients, especially within the 30 days after discharge from the hospital. In this paper, a statistical model was proposed to predict the readmission risk of COPD patients within 30-days by monitoring their physical activity (PA) in daily living with accelerometer-based wrist-worn wearable devices. This proposed model was based on our previously reported PA models for activity index (AI) and regularity index (RI) and it introduced a new parameter, quality of activity (QoA), which incorporates previously proposed parameters, such as AI and RI, with other activity-based indices to predict the readmission risk. Data were collected from continuous PA monitoring of 16 COPD patients after hospital discharge as test subjects and readmission prediction criteria were proposed, with a 63% sensitivity and a 37.78% positive prediction rate. Compared to other clinical assessment, diagnosis, and prevention methods, the proposed model showed significant improvement in predicting the 30-day readmission risk.

## 1. Introduction

Chronic obstructive pulmonary disease (COPD) ranked no. 3 in the list of top 10 causes of deaths in 2016 according to the report [1] from the World Health Organization (WHO) on 24 May 2018. Indeed, COPD claimed 3.0 million lives in that year, while lung cancer caused 1.7 million deaths. Moreover, COPD has the highest readmission risk within 30 days among all chronic diseases after patients were discharged alive from hospitals [2]. Readmission into the hospital usually means rehospitalization as a consequence of patients’ worsening health conditions after discharge from hospital. COPD patients not readmitted into the hospital when necessary may jeopardize the patients’ health conditions and this may even lead to mortality. However, too frequent readmission would consume a lot of the available medical resources: either the medical care human resources or the equipment, medications, and treatments, which are necessary to improve patients’ health conditions. Hence, a methodology to predict readmission of COPD patients after discharge from hospital, especially within 30 days, is highly desired. Therefore, in this way, COPD patients can have changes in their diagnosis and treatment before their symptoms actually become worse and it also prevents consuming too many medical resources when a COPD patient is readmitted.

The existing diagnosis methods that are adopted clinically by physicians, such as the pocket guide to COPD diagnosis, management, and prevention–Global Initiative for Chronic Obstructive Lung Disease (GOLD) 2019 [3], Body-mass index, airflow Obstruction, Dyspnea, and Exercise (BODE) index [4], modified Medical Research Council (mMRC), COPD assessment test (CAT), 6-minute walking distance, and measurement of forced expiratory volume in one second (FEV1), might be able to assess the severity of the patient’s condition but these cannot predict the readmission risk of COPD patients especially within 30 days. Moreover, these tests might not even be able to reduce the readmission rate of the discharged COPD patients. Tsui et al. [5] performed extensive studies on risk factor assessment for the readmission of COPD patients after implementing the GOLD guidelines and their results indicated that the readmission rate for acute exacerbation of COPD (AECOPD) remains high after the implementation of GOLD treatment guidelines.

It is a well-known fact that once the health conditions of COPD patients worsen, their ability to perform physical activities (PAs) usually degrades significantly due to their physical condition. In 2007, Bahadori and Fitzgerald [6] investigated a number of potential modifiable factors or parameters that were independently associated with a higher risk of COPD exacerbation requiring readmission to the hospital and physical activity was one of the important factors. Trost et al. had shown the utility of artificial neural network (ANN) to predict activity type, i.e., level of activity and energy expenditure which are directly related with physical activity [7]. Osman et al. have shown that quality of life scores may predict readmission for COPD or death within 12 months of an original admission [8]. Pitta et al. [9] concluded that even acute exacerbations (AEs) have a negative impact on various aspects of the progression of COPD, however, efforts to enhance physical activity should be among the aims of disease management during and following the AE periods. Still, these researches have not discussed the potential rehospitalization risk, which means early signs of rehospitalization or emergency room visit based upon COPD risk factors are difficult to predict.

Indeed, lack of PA is an important risk factor for COPD patients and it may cause associated morbidities like cardiovascular disease, hypertension, diabetes mellitus, obesity, stroke, cancer, and osteoporosis. Past research has shown that wrist-worn accelerometer-based devices could successfully be employed for the assessment of physical activity in COPD patients and might provide patients with the information needed to maintain a definite level of PA in daily life [10,11]. Small size, low-cost, accelerometer-based wrist-worn devices integrated with Internet-of-Thing (IoT) can provide valid and useful estimates of within-person differences in metabolic equivalent level over three-hour periods in patients with COPD [12].

Through literature research, we were motivated to make use of accelerometer-based wrist-worn devices, such as an actigraphy device, for continuously recording and monitoring the PA of COPD patients discharged from the hospital to predict the potential readmission of COPD patients especially within 30 days. Hence, in this study, PA data of the COPD patients discharged from hospital were collected with the actigraphy device and the collected acceleration data were analyzed based on the previously proposed models [13]. Based on these models, parameters, such as activity indices (AIs) and regularity indices (RIs), were calculated. Furthermore, quality of activity (QoA) was proposed in this study to reflect the observation of “Act it Right would be more Important than Act it More”. Through the trend analysis of patients’ activities based on these parameters, the possibility of readmission for the discharged COPD patients were generated. This paper is organized as follows: in Section 2, the materials used and subjects involved in the Institutional Review Board (IRB) testing of this study are described. The methods used for the trend analysis and the developed prediction criteria are also explained in this section. Section 3 presents the results of the prediction by comparing the dates of patients’ discharge with actual emergency room visiting and even rehospitalization. Finally, in Section 4, we discuss several factors that might impact our prediction and, in the last section, we draw some conclusions for the direction of future works.

## 2. Materials and Methods

This study was reviewed and approved by the institutional review board (IRB) of the Chang Gung Memorial Hospital, Taiwan, R.O.C. Originally, 18 COPD patients discharged from hospital participated in this study. They were asked to wear an accelerometer-based actigraphy device, i.e., GeneActiv, from Activinsights Ltd., Huntingdon, UK, on their wrists 24 h a day to continuously monitor the activity level in their daily life at home after being discharged. They were scheduled to go back to the hospital for a routine check-up every 2–4 weeks. On return to the hospital, the actigraphy device was removed and replaced with another fully charged device. After actigraphy devices were removed from patients, the stored data inside the devices were downloaded and analyzed.

Table 1 lists some statistical information about this study. Since patients’ data were compared with themselves (self-referential) in the trend analysis, gender and age of the patients were irrelevant in this study. Hence, this information is not listed in the table. A total of 18 patients participated in the test. However, there were 2 patients who either had too short a duration of valid data for the trend analysis or who withdrew from participation in the middle of testing and, hence, their data were not included in the study. So, only 16 patients’ data were analyzed in this study. The longest period of days for patient participation in the test was more than 1 year. However, for some reasons, such as device failures, forgetting to wear the device after the patient removed the device for a short period of time, for example, taking a shower, etc., only valid data were analyzed in this study. In the data analysis, when the actigraphy device was removed from the patient’s body for more than one hour then the data of the day was considered as invalid. For those patients that participated in the test, there was a total of 21 incidents of emergency room visits (ER visiting) or rehospitalization. These 21 events became the check points to validate whether the prediction made for the 30-day readmission was correct or not.

### 2.1. Automation of Data Processing

The wrist-worn device equipped with an accelerometer was set to record the data at a sampling rate of 20 Hz. Under this sampling rate, there were 1,728,000 packets of measured data in a whole day, i.e., 24(h) × 60(min) × 60(s) × 20(Hz). The downloaded data from the removed device were stored as a single file containing multiple days of data between two consecutive routine check-ups. For a typical 2–4 weeks of duration, the sizes of .csv files usually were around 2–3 Gbytes.

To reduce the amount of raw data for trend analysis to predict readmission within 30 days, raw data were converted into minute-wise activity indices (AIs) and the day-to-day regularity indices (RIs) were calculated according to the models proposed in [13]. It would have taken a tremendous amount of effort and tedious work to process the raw data manually.

**Figure 1:**
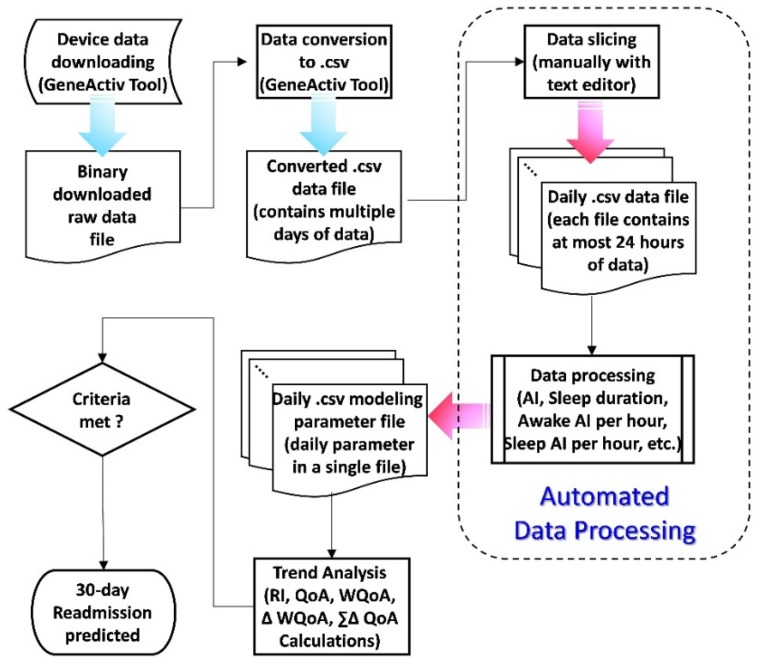
The flow chart of data processing for this study. (RI stands for “Regularity Index”, QoA stands for “Quality of Activity”, WQoA stands for “Weighted Quality of Activity”, ∆WQoA is the difference between two consecutive WQoAs, and ∑∆QoA is the summation of the difference of QoA within certain period).

Figure 1 shows the flow chart of the data processing methodology used for this study. The raw data were downloaded from the actigraphy device in binary format, i.e., “.bin” file, which contained all the data stored in this device during the testing period. Then, it was converted into the comma separated values (.csv) file format using the software tool, i.e., the GeneActiv tool, provided by the device manufacturer. The .csv file can be processed later with a spreadsheet program (such as Microsoft Excel). Since the file contained multiple days of the measured acceleration data, it had to be sliced into multiple .csv files so that each file contained at most a single day’s data (noon to noon: from 12:00 of one day to 12:00 of the next day) for further data processing. From these files containing at most 24 h of data each, data processing was performed and the related parameters, such as minute-wise activity indices (AIs), sleep duration detection, awake AI per hour, sleep AI per hour, were generated and written into separated .csv files.

After that, the day-to-day regularity indices (RIs) as well as other modeling parameters to be discussed later for the trend analysis could be processed. Finally, based on the results of the trend analysis, if these parameters met the criteria for potential readmission within next 30 days, then a prediction and warning were raised.

If all of the data processing steps, as shown in Figure 1, were performed manually, it would have been very time consuming and required tremendous amount of tedious human effort. It is worth mentioning that the converted .csv data file was too large to be processed by most commonly available text editor and spreadsheet tools for any data processing task. Hence, efforts were made to develop an automated data processing program that took the original .csv data file, which contained multiple days of data, as an input file and sliced it into separate different data files containing 24-hr day-wise data. Then, the PA related parameters, such as minute-wise activity indices (AIs), sleep duration detection (in minutes), awake AI per hour, sleep AI per hour, as described in [13], were generated and written into separate .csv files as the output of the automated data processing tool.

The developed tool reduced tremendous amount of time and effort for the data processing and, after that, we could investigate how the data should be further processed and which of the varying trends of the activity-based parameters could possibly indicate the readmission within next 30 days.

### 2.2. Quality of Activity (QoA): Quality of Activity is More Important than Quantity of Activity

As described in [13], the motivation to quantify the regularity of activity performed in daily life came from the observation of a patient who had an extremely irregular daily activity and, unfortunately, the patient went to hospital for an ER visit the next day. We further investigated this patient’s data and found that his total AI of that day, i.e., the summation of 1440 minute-wise AI, was on the same level compared to when he was just discharged from hospital. The only significant changes were his day-to-day daily living regularities.

This means, it is not quite enough to consider the amount or level of activities, i.e., total AI, performed by the subject only. Even with a high amount of activity performed by the patient on the day, it does not necessarily indicate that the patient would be in good physical condition. Skene et al. [14] found that shift workers, whose schedules are misaligned relative to their suprachiasmatic nuclei (SCN) circadian pacemaker, are at an elevated risk of metabolic disorders. Hence, this observation supports the idea that “Quality of Activity is More Important than Quantity of Activity”. Therefore, with the AIs or RIs parameters alone, these wouldn’t be able to reflect truly the actual physical condition of the patients. These parameters have to be combined into a new parameter that can represent the combined effects of physical activity as well as the regularity of physical activity in the daily living to reflect the subject’s physical condition. Thereby, the quality of activity (*QoA*) was proposed in this study for this purpose. The *QoA* is calculated as in Equation (1):

*QoA* = Total_AI*(1 + *RI*), (1)

where *Total_AI* is the summation of the minute-wise AIs of the day and *RI* is the regularity index of the day compared with the day before.

### 2.3. Modeling of the Activity-Based Trend Analysis to Predict 30-Day Readmission for COPD Patients

To predict if the COPD patient discharged from the hospital could possibly be readmitted within the next 30 days based on the patient’s amount of daily physical activity performed at home, we compared the changes in the quality of physical activities performed by the patients themselves continuously right after the patients had been discharged.

This kind of self-referential trend analysis was necessary because the amount of activity performed by each individual varies a lot from person to person even when they are all in good physical condition. For this purpose, the following models and parameters were calculated for the trend analysis and were considered as the criteria if the prediction for readmission would be raised or not:

(2) $$∆{\mathrm{QoA}}_{i}=({\mathrm{QoA}}_{i}-{\mathrm{QoA}}_{i-1})/{\mathrm{QoA}}_{i-1}*100,$$

*WQoA_i_* = 0.4*QoA_i_ + 0.3*QoA_i__−1_ + 0.2*QoA_i__−2_ + 0.1*QoA_i__−3_, (3)

(4) $$∆{\mathrm{WQoA}}_{i}=({\mathrm{WQoA}}_{i}-{\mathrm{WQoA}}_{i-1})/{\mathrm{WQoA}}_{i-1}*100,$$

(5) $$\sum ∆{\mathrm{QoA}}_{i}=\sum_{j=0}^{j=6}∆QoA_{i-j},$$

where, in Equation (2), ∆*QoA_i_* represents the percentage change of *QoA* on day *i* as compared to the previous day, i.e., day *i* − 1. In Equation (3), *WQoA_i_* is the weighted running average of the recent 4 days’ (starting from day *i*) *QoA*. In Equation (4), ∆*WQoA_i_* represents the percentage change in *WQoA* on day *i* compared with the day before, i.e., day *i* − 1, and $\sum ∆QoA_{i}$ is the total percentage of *QoA* changes in the recent 7 days starting on day *i*.

### 2.4. Criteria for the Prediction of an ER Visit or Readmission within 30 Days

**Figure 2:**
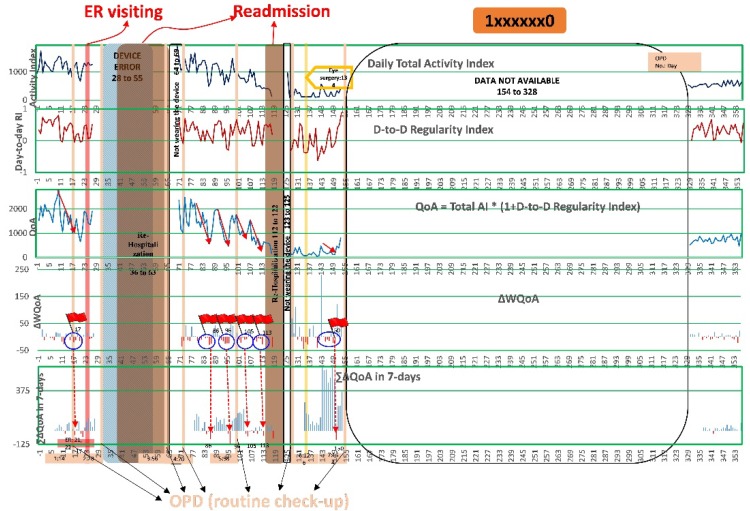
The trend of Activity Index (AI), Regularity Index (RI), Quality of Activity (QoA), Difference of Weighted Quality of Activity (∆WQoA), and Summation of the difference of Quality of Activity (∑∆QoA) for patient (ID: 1xxxxxx0) after discharged.

Figure 2 shows the PA data chart of one patient (ID #1xxxxxx0) with daily total AI, RI, QoA, ∆WQoA, and ∑∆QoA beginning the day that the patient was discharged from hospital. The *X*-axis of the chart is the *i*th day since discharged. On the days the patient went back to the hospital for routine check-up (OPDs), ER visits and rehospitalizations are clearly highlighted on the chart with different colors. Even though there were 175 days of data, entire data are not available due to the patient not wearing the device in the middle of the test period, it could still be observed that there were obvious downward trends (marked with arrows in red color) of QoA within 30 days before ER visits or rehospitalizations. On the charts of ∆WQoA and ∑∆QoA, red bars stand for when the values decreased and blue bars stand for the when values increases. It could also be observed that there were several continuous drops of ∆WQoA and a significant dropping of ∑∆QoA within 30 days before ER visits or rehospitalizations.

Even though Figure 2 only shows a single patient’s data, similar trends were also observed from other patients’ data. Based on these observations, the prediction criteria were set to:

- More than 4 days in the past 7 days of ∆WQoA dropping;
- ∑∆QoA drops more than 30% on that day.

On the day, when both prediction criteria were met, the prediction of an ER visit or rehospitalization within the next 30 days was found to be true.

## 3. Results

The sensitivity (true positive rate), positive predictive rate, false negative rate, and false positive rate were calculated based on the prediction criteria proposed in Section 2.4.

### 3.1. Performance Evaluation of the Prediction

Based on the prediction criteria proposed in Section 2.4, the 16 patients whose data was analyzed in this study, their actual clinical status (number of ER visits and rehospitalizations) was compared with the correctness of the predictions, as summarized in Table 2.

In Table 2, the column titled “No. of ER visits or rehospitalizations—Actual” is the actual number of ER visits or rehospitalizations of that specific patient. The column titled with “No. of ER visits or rehospitalizations—Predicted” is the number of predicted ER visits or rehospitalizations within the next 30 days. There might be multiple predictions for a single ER visit or rehospitalization event. For these predictions, when there was an ER visit or rehospitalization within the next 30 days, then the prediction was considered to be correct and the number of these correct predictions is listed in the column titled “Truly Predicted”. If nothing happened, i.e., neither an ER visit nor rehospitalization, within the next 30 days when a prediction was made otherwise, then the prediction was considered to be a false alarm. The number of these false predictions are counted in the column titled “Falsely Predicted”. The summation of the numbers listed in the columns of “Truly Predicted” and “Falsely Predicted” should be equal to the number listed in the column of “No. of ER visits or rehospitalizations—Predicted”. The number of actual ER visit or rehospitalization events that were not predicted before 30 days were counted as “Not Predicted”. The spreadsheet of the patients’ data with the generated predictions is included as Supplemental Materials.

**Figure 3:**
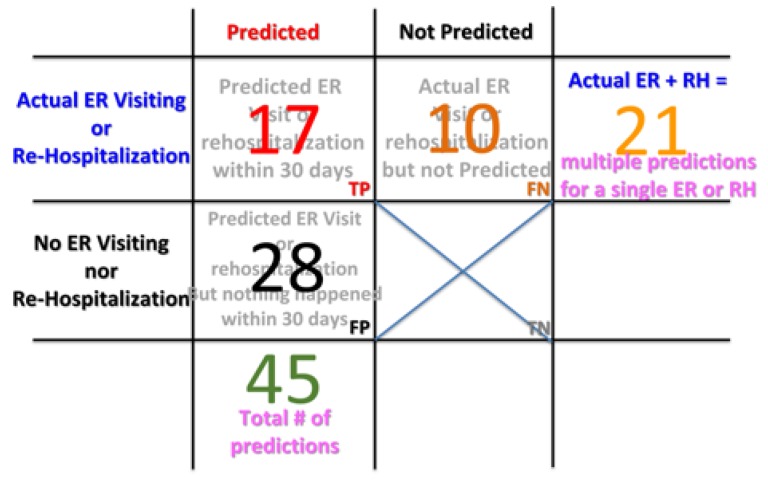
The true/false positive/negative charts of the predictions. (RH stands for “ReHospitalization”, TP stands for “True Positive”, FN stands for “False Negative”, FP stands for “False Positive”, and TN stands for “True Negative”).

As listed in Table 2, there were a total of 45 predictions made. Out of these prediction, 17 of them were considered to correct predictions because, as per clinical status record, patients actually had ER visits or rehospitalization, which was listed as the case of true positive (TP), as shown in Figure 3. Twenty-eight of these predictions were false alarms and were considered to false positives (FP). In the IRB test period, there were 10 patient ER visits or rehospitalizations prior to 30 days that were not predicted. Hence, there were considered to be false negative (FN) cases. Note that, even though there were 21 actual ER visits or rehospitalizations in total, there could, however, multiple predictions could be generated for a single ER visit or re-hospitalization event. Therefore, the number of TP and FN do not add up to 21. In this study, there were no true negative (TN) cases counted, as seen in Figure 3, since there is no way to count how many times that no prediction was made and the patients were actually in good condition without an ER visit or rehospitalization.

Based on Figure 3, the statistical measures of the performance of the proposed COPD 30-day readmission were calculated as follows:

- Sensitivity (true positive rate), i.e., percent of actual ER or ReHospitalization (RH) predicted before 30 days, = TP/(TP+FN) = 62.96%;
- Precision (positive predictive rate), i.e., percent of predictions that were considered to be correct, = TP/(TP+FP) = 37.78%;
- Miss rate (false negative rate), i.e., percent of actual ER or RH but not predicted before 30 days, = FN/(TP+FN) = 37.04%;
- False discovery rate, i.e., percent of predictions that were considered to be false alarms, = FP/(FP+TP) = 62.22%.

Since there were no TN cases in this study, the specificity, negative predictive rate, and fall-out rate (i.e., false positive rate) were ruled out and not calculated.

### 3.2. Evaluation of the Prediction Criteria

The prediction criteria were proposed in Section 2.4. and their performance was evaluated in Section 3.1. We then analyzed how the model performance would change if the prediction criteria were loosened or made stricter. In this subsection, the performance evaluation of the looser and stricter prediction criteria are evaluated to judge if the criteria proposed in Section 2.4 were appropriate or not.

Hereby, the prediction performance with two other criteria are tested and evaluated.

1. Stricter criteria
  - More than 4 days in the past 7 days of ∆WQoA dropping;
  - ∑∆QoA drops more than 30% on that day;
  - Three continuous days of ∆WQoA dropping before (and on) the day that the prediction is raised.
2. Looser criteria
  - More than 3 days in the past 7 days of ∆WQoA dropping;
  - ∑∆QoA drops more than 25% on that day.

The first prediction criteria are considered to be stricter than the ones first proposed since not only did the two original criteria need to be met, but also there should be more than (or equal to) 3 continuous drops of ∆WQoA before the day that the prediction can be generated. The second prediction criteria are considered to be looser since it only requires 3 days of ∆WQoA dropping in the past 7 days and also 25% of ∑∆QoA dropping on the day that the prediction can be generated.

Based on these two criteria (loosened and stricter), the statistical measurements of their prediction performance are compared in Table 3 along with the statistical measures of the original proposed criteria. As expected, when the prediction criteria were loosened, the number of successful predictions increased as did the number of false predictions. Hence, the sensitivity, i.e., the true positive rate, increased to 72.7%. However, the false discovery rate also increased to 63.7%. When the criteria become stricter, the number of false predictions decreased but the number of successful predictions also decreased. Hence, the sensitivity dropped significantly to 37.5% and the false discovery rate only decreased slightly to 59.1% from 62.22% of the original proposed criteria.

From this prediction performance comparison, it can be inferred that the proposed criteria in result in the optimal performance as compared to the loosened or stricter criteria.

## 4. Discussion

Even though the false discovery rate was as high as 62.22%, i.e., a high number of false positive predictions, the situation is still acceptable for the purpose of medical prevention. In some cases, patients might actually suffer from illness but they tend to not go to the hospitals. Under this circumstance, prompt phone calls from the health supervisor of the clinical institution asking about the health condition of the patient will be highly appreciated.

There were also couple of other reasons for the high number of false positive predictions in this study. The first one is that no clinical status (ER visit or rehospitalization) was recorded after the predictions were raised. In this study, 7 out of the 28 were considered to be false positives because there were no clinical records right after 30 days of predictions had been generated. The second reason is that the patients might have been engaging in rehabilitation regularly during the IRB testing period. Rehabilitation may actually improve the physical condition of the patients after the prediction for 30-day readmission so that these predictions were also considered to be false positives. The last, but not the least, reason is that, even as a prediction was generated, if the patient had a routine hospital check-up within the next 30 days, the physician might change the medication or treatment plan for the patient so that the patient’s physical condition improved and no ER visit nor rehospitalization were required. In this IRB study, 17 out of 28 false positive predictions, patients actually had the routine outpatient clinic (OPD) visit within the next 30 days of the readmission predictions. This may also be the reason why there was no ER visit or rehospitalization within the next 30 days of prediction and hence the prediction was considered to be a false positive one.

It may also be criticized that the missed prediction rate is high, reaching as high as 37.04%. There were also some situations that would result in the high false negative cases (10 in this study), i.e., there were actual ER visits or rehospitalization but without any prediction prior to the 30 days before. The first one would be that the monitoring data were not continuous in the 30 days prior to when the patients were having ER visits or rehospitalization. In this situation, the algorithm proposed might miss the data necessary for the prediction. In this study, 7 out of the 10 false negative cases belonged to this situation. The second reason is that when patients were having ER visits or rehospitalization, the actigraphy device was removed during this period in the hospital. Therefore, if the patient had an ER visit or rehospitalization within 7 days after being discharged, then there was no way to predict the new ER visit or rehospitalization of the patient. One out of the total 10 mispredicted ER/RH events belongs to this situation.

A previous study emphasized the clinical index, including age, prior missed outpatient appointments, length of hospital stay, and comorbidities, to predict readmission [15]. However, the most valuable index is still unknown. Intensive outpatient monitoring, evaluation, and follow-up after discharge are needed to help prevent return to the emergency department and readmission to the hospital for a variety of clinical complaints [16]. Physical activity is reported to be a predictor of 30-day hospital readmission after a discharge for clinical exacerbation of COPD [17]. However, currently, there is inadequate evidence to endorse specific intensive outpatient monitoring for physical activity to reduce readmission among patients with COPD. Therefore, our study provided a new potential device to improve patient outcome and save valuable hospital resources. Also, it could be used by the patients at home after discharge from the hospital.

## 5. Conclusions

In this study, statistical models for trend analysis, algorithm for data processing, and readmission prediction criteria were proposed for COPD patient readmission within 30 days after hospital discharge using accelerometer-based wearable devices, i.e., an actigraphy device, to monitor the amount of activities performed at home. A novel parameter, i.e., quality of activity (QoA), was proposed as a combined index that takes both amount of activity (AIs) performed and regularity of daily activity (RIs) into consideration. In the trend analysis of the monitored data, the prediction criteria only used the most recent 7 days of data. This means that, after the patient wearing the device has been discharged for 7 days, the algorithm starts to predict if the patient will be readmitted or not.

Based on the continuous PA monitoring of 16 COPD patients discharged from hospital, with the proposed prediction criteria, a 63% sensitivity with a 37.78% positive prediction rate was achieved. Even though the positive prediction rate was only 37.78%, the physicians were amazed with the performance of the prediction. They commented that, even if 9 out of 10 predictions were false alarms and only 1 actual ER visit or rehospitalization was predicted, it could still even save a patient’s life.

In the future, this prediction methodology could be implemented as a platform that uses smart wrist-band monitoring to collect the COPD patients’ physical activity data after they are discharged from hospital. The measured raw data will then be converted into the minute-wise AI parameter and then these data would be transmitted to a health-cloud through smart phones. The trend analysis of the patient’s activity and readmission prediction will be generated on the cloud. When a positive prediction is made, it will be pushed to the clinical institution and the physician and personal health supervisor will be informed. Then the personal health supervisor of the patient can give the patient a call to show concern about the physical condition of the patient and the patient can be requested to be rehospitalized, if required. In this way, the COPD patient need not worry about multiple false positive predictions if the predictions are sent to the patients directly. Instead, the patients will appreciate the phone calls from a personal health supervisor and feel that they still have been taken care of by the clinical institution even though they have already been discharged from the hospital.

## Figures and Tables

**Table 1:** Statistic items in the test.

Statistic Items	Tested	Analyzed
No. of patients	18	16
Total no. of days	3565	3298
Total no. of days with valid data	1937	1761
Longest no. of days of testing	448	448
Shortest no. of days of testing	16	16
Longest no. of days of testing with valid data	251	251
Shortest no. of days of testing with valid data	16	16
Average no. of days in testing per patient	198	206
Average no. of days in testing per patient with valid data	108	110
No. of ER visits or rehospitalization events	21	21

**Table 2:** Summary of readmission prediction and actual number of ER visits or rehospitalizations.

COPD Patient	No. of ER Visits or Rehospitalizations		Truly Predicted	Falsely Predicted	Not Predicted
	Actual	Predicted			
1	0	3	0	3	0
2	2	1	1	0	1
3	1	2	2	0	0
4	0	4	0	4	0
5	0	1	0	1	0
6	0	0	0	0	0
7	0	0	0	0	0
8	0	0	0	0	0
9	3	7	1	6	2
10	1	3	0	3	1
11	0	0	0	0	0
12	3	5	3	2	1
13	5	7	6	1	3
14	0	3	0	3	0
15	0	4	0	4	0
16	6	5	4	1	2
**Total**	21	45	17	28	10

**Table 3:** Statistical measurements of the three different prediction criteria (loosened, proposed, and stricter).

Numbers	Loosened	Proposed	Stricter
no. of successful predictions (TP)	24	17	9
no. of false predictions (FP)	42	28	13
no. of ER/RH events not predicted (FN)	9	10	15
Sensitivity—TP/(TP+FN) (%)	72.7	62.96	37.5
False discovery rate—FP/(TP+FP) (%)	63.7	62.22	59.1

## Supplementary Materials

The spreadsheet of the patients’ data with the generated predictions is available online at https://www.mdpi.com/1424-8220/20/1/217/s1.
