# Supplementary figures and images for: Prediction of 30-Day Readmission for COPD Patients Using Accelerometer-Based Activity Monitoring
Source: Sensors (Basel). 2019 Dec 30;20(1):217. doi: 10.3390/s20010217 (PMC6982816; doi:10.3390/s20010217)

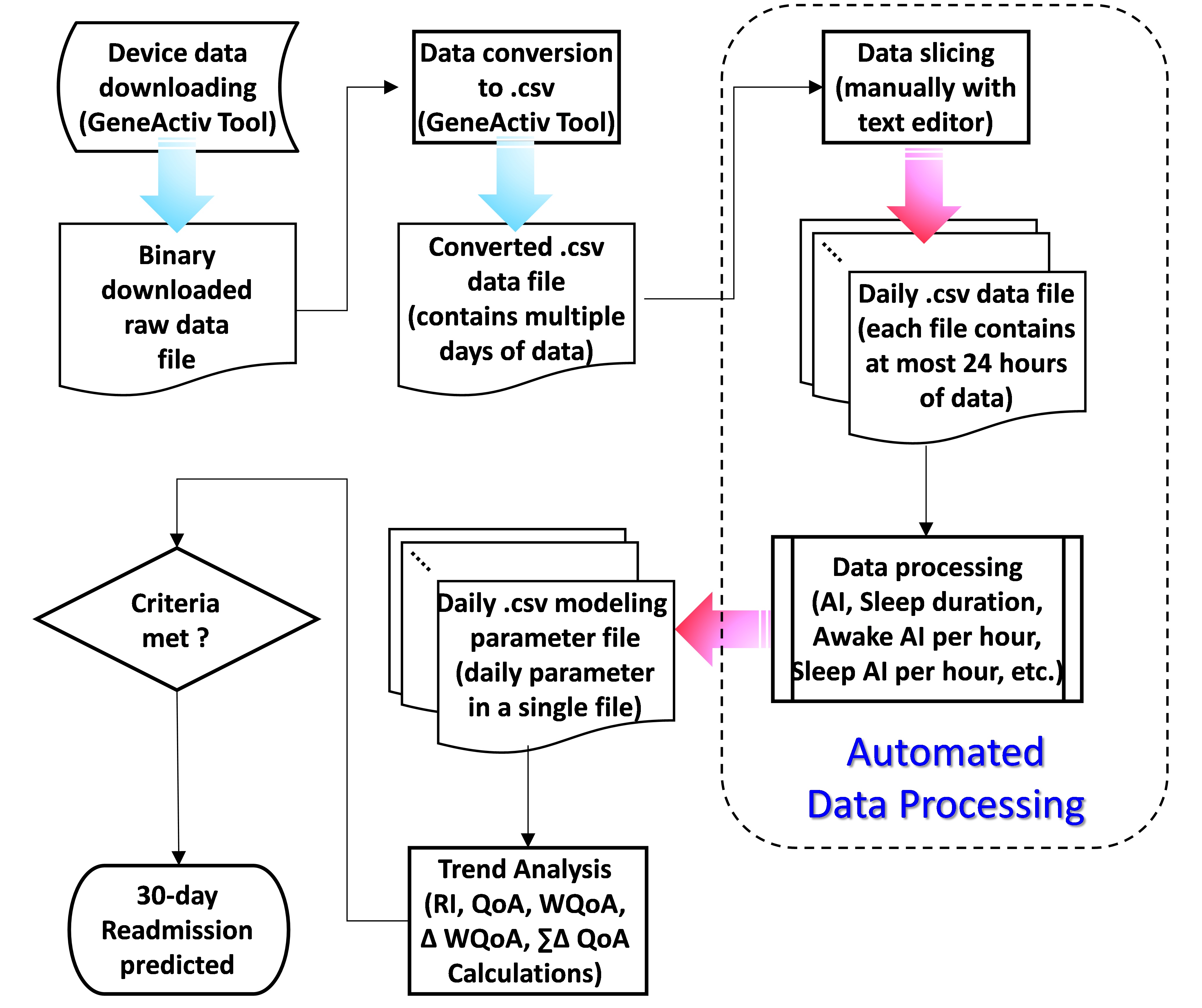

Supplement: Supplementary file 1 [file sensors-20-00217-s001.zip › Figures/fig1_cutted.jpg]

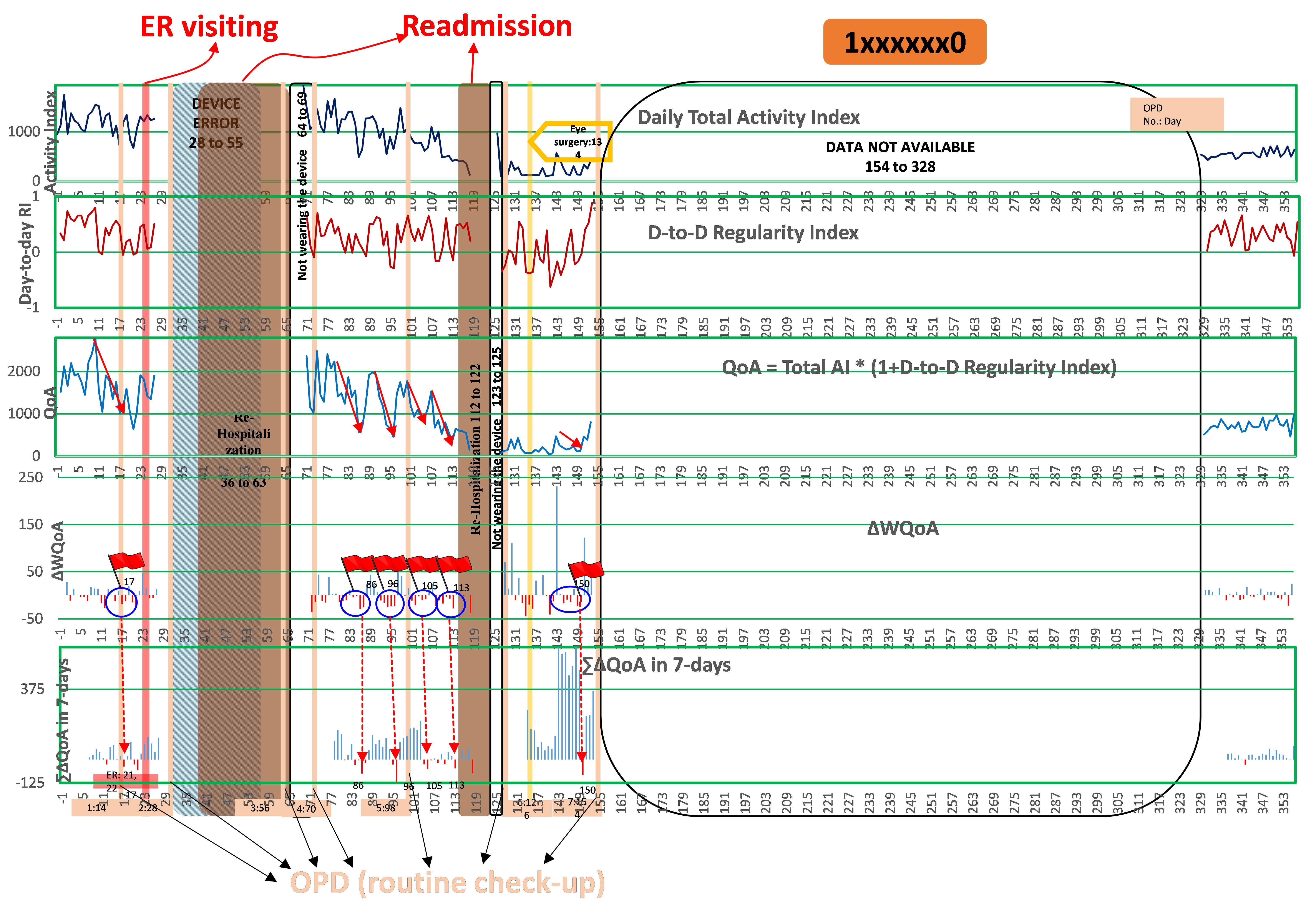

Supplement: Supplementary file 1 [file sensors-20-00217-s001.zip › Figures/fig2_cutted.jpg]
